# Effects of Laser Scanning Strategy on Bending Behavior and Microstructure of DP980 Steel

**DOI:** 10.3390/ma17102415

**Published:** 2024-05-17

**Authors:** Wenbin Dong, Yajing Zhang, Le Bao, Kyoosik Shin

**Affiliations:** 1 Department of Mechanical Engineering, Anhui Science and Technology University, Chuzhou 233100, China; 2 Department of Mechatronics Engineering, Hanyang University, Ansan 15588, Gyeonggi-do, Republic of Korea

**Keywords:** accumulated line energy density, peak temperature, bending angle, microstructure evolution, grain refinement

## Abstract

Laser bending is a kind of cumulative forming technology and bending efficiency is one of its most important indexes. This study investigates the bending behavior and the microstructure of DP980 steel plates under different laser scanning strategies, using an IPG laser system. Two sets of experiments varied the accumulated line energy density (AED) by altering the laser scanning velocity and number of scans. The results show that, for the single laser scanning process, the bending angle of the plate increases with AED, due to a larger temperature gradient through the thickness direction; however, this relationship is nonlinear. A higher AED led to a sharper initial increase in bending angle, which then plateaued. Under the same AED conditions, the bending angle of the plate undergoing multiple laser scans increases by at least 26% compared to the single one, due to the microstructure changes. It is revealed that the bending efficiency is affected by both the AED and the resultant microstructure evolution in the DP980 steel. Higher AED values and appropriate peak temperatures facilitate better bending behavior due to the formation of uniform martensite and grain refinement. Conversely, excessive peak temperatures can hinder bending due to grain growth.

## 1. Introduction

Laser bending is a fascinating and innovative technique in the realm of material processing, particularly in the precision forming of all kinds of metals, composites, and even ceramics [1,2]. This technique, leveraging the concentrated heat of a laser to induce deformation, offers a non-contact, tool-free method for bending materials, thus minimizing physical stress and tool wear. It is well suited for prototypes and custom designs and is especially beneficial in industries where customization and precision are critical, such as aerospace, automotive, and medical devices [3].

Due to the small bending angle per laser scanning and the inescapability of edge effect [4,5], a considerable part of previous research has focused on the laser bending efficiency and precision. Many researchers have studied the effects of laser parameters on the bending angles of the plates and have explored the optimization of operational parameters, such as Fu et al. [6], Annamaria et al. [7], and Nejad et al. [8]. Liu et al. [9] and Safari [10] contributed to the methodology by comparing different laser irradiating schemes to improve the forming precision. Moreover, Lambiase et al. [11] analyzed productivity in the multi-pass laser forming of thin stainless steel sheets, providing valuable perspectives on forming efficiency. Shi et al. [12] and Guo et al. [13] proposed a new strategy to enhance the forming efficiency and surface quality of the plate by preloading at a lower heating temperature. Kalvettukaran et al. [14] and Yadav et al. [15] presented an innovative approach to improve the forming competency by forced cooling. Furthermore, Abolhasani et al. [16] introduced a double raster laser scanning strategy, an innovative approach for the rapid die-less bending of 3D shapes, showcasing the potential for further technological advancements in laser forming. Miao et al. [17] provided insights into the spring-back deformation of CF/PEEK thin angled laminates by laser-assisted forming, which is a crucial consideration in achieving dimensional accuracy in complex components. These studies collectively highlight the influencing factors of laser bending angle and proposed strategies to improve the forming efficiency and precision.

Laser bending is a thermoforming process and the microstructure of the material may change during processing, which will affect the properties of the material. More and more research focusing on the microstructure and properties of the material has been published in recent years. Ramos et al. [18] investigated the microstructural changes and hardness variations of a laser bent Al-2024-T3 alloy. They revealed that the microstructure and hardness change considerably with different AED levels. Liu et al. [19] studied the microstructure and properties of a laser bent Ti-7Al-2Zr-2Mo-2V material and proposed that the critical value of AED is 40 J/mm^2^. Knupfer and Moore [20] assessed the mechanical and metallurgical properties of low-carbon steel and an aluminum alloy after laser forming. They found the increased hardness temperature range of the low-carbon steel and the reduced hardness temperature range of the aluminum alloy. Abazari et al. [21] delved into the microstructural and mechanical properties such as tensile, fatigue, and hardness evolution in SUS430/C11000/SUS430 composites and provided some suggestions for enhancing the properties of the material. Shi et al. [22] explored the microstructure and mechanical behaviors of laser-formed Nb-23Ti-15Al alloys. They provided insights into achieving nearly defect-less structures with refined microhardness and fracture toughness, showcasing the ability of laser forming to enhance material properties for high-performance applications. Guo et al. [23] proposed an innovative Applying Baffles Method (ABM) based on thermal stress forming, demonstrating a significant increase in the bending angle and grain refinement of Q235 steel. This advancement marks a critical step in broadening the applicability of laser forming in areas with high mechanical performance requirements. The authors of [24] investigated the effects of laser forming on the yield and tensile strength, as well as the microhardness, of DP980 steel, illustrating the method’s effectiveness in enhancing key mechanical properties.

In brief, the previous studies either highlighted the forming efficiency and precision, or considered the performance, of the laser-formed material. They are pivotal for the development of laser forming technology. However, as laser bending is a complex thermo-mechanical coupling process, if a strategy can be found to improve the forming efficiency, while improving the material properties, it will be conducive to the integration of deformation and the performance of the laser bending technology. The current study focused on exploring the relationship between process parameters, microstructure evolution, and the bending behavior of the material. This understanding will enable scientists and engineers to make more informed decisions when designing laser bending processes, leading to improved forming efficiency and material properties.

## 2. Materials and Experiments

### 2.1. Materials

**Figure 1:**
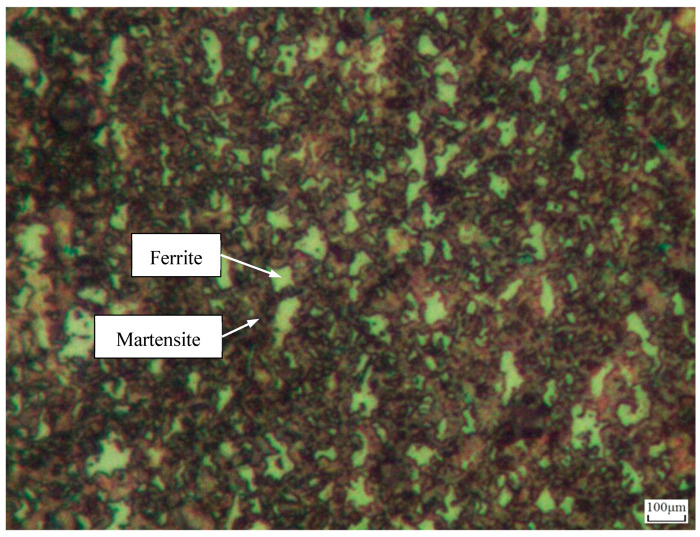
Microstructure of DP980 steel.

DP980 steel is a kind of high-strength steel whose average tensile strength is not less than 980 MPa. It is widely used in the automotive industry, especially in automotive body structures, chassis, suspension systems, and other key components. It can effectively improve the safety and durability of the automobile and reduce the production cost. DP980 steel is obtained via the heat treatment of mild steel. The main chemical component of DP980 steel is Fe, with other components being listed in Table 1 [25]. Its microstructure mainly consists of ferrite and martensite, as shown in Figure 1. Among them, ferrite is the matrix phase and martensite is the strengthening phase. The presence of martensite produces DP980 steel with a high strength and good plasticity [26]. The tensile strength of DP980 steel is around 1 GPa, while the elongation is not less than 10% and the microhardness is larger than 300 HV_0.2_ [24].

### 2.2. Laser Bending Experiment

**Figure 2:**
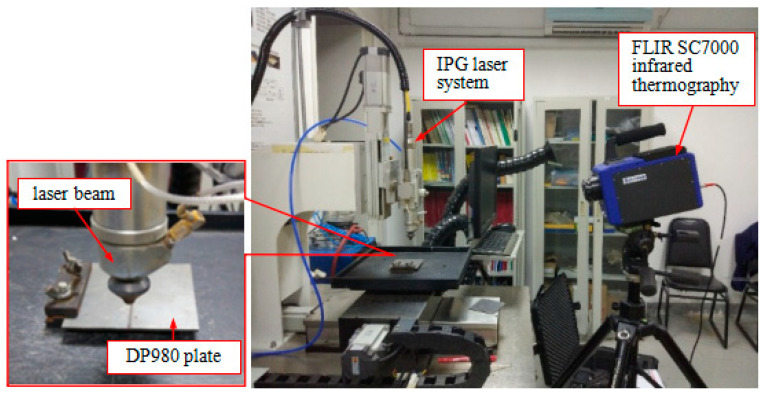
Laser bending experiment setup.

A laser system from IPG company was employed for the laser bending experiment. The parameters of the system are listed in Table 2. A rectangular specimen with a length and width of 80 mm and a thickness of 1.8 mm was fixed at one side and was scanned by the laser beam through the center line of the top surface, as shown in Figure 2. The defocusing distance of the laser beam is 10 mm. The temperature of the material is recorded using FLIR SC7000 infrared thermography (FLIR Systems, Wilsonville, OR, USA) and the bending angle is measured using a KEYENCE-KS1100 3D surface profiler (KEYENCE, Osaka, Japan).

During the laser scanning process, the DP980 plate bent towards the laser beam due to the thermal stress induced by the laser energy. Accumulated line energy density (AED) is usually used to measure the laser energy absorbed by the plate, which can be expressed as in Equation (1) [19]:

(1) AED=ηnP/dv

where η, n, P, d, and v denote the absorptivity, number of scans, laser power, spot diameter, and scanning velocity, respectively. Among them, η has a value of 0.75 [27] and d has a value of 2.5 mm. In order to explore the effects of the laser scanning strategy, two groups of experiment were carried out, as listed in Table 3 and Table 4.

## 3. Results and Discussion

### 3.1. Bending Behavior

**Figure 3:**
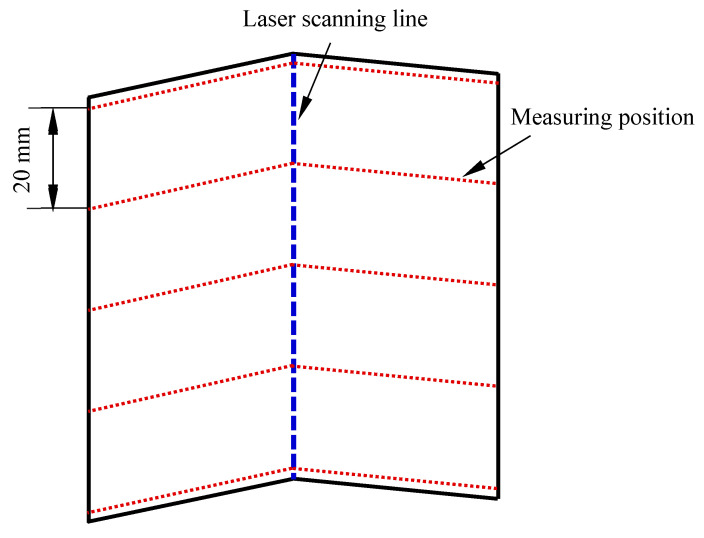
Bending angle measurement diagram.

**Figure 4:**
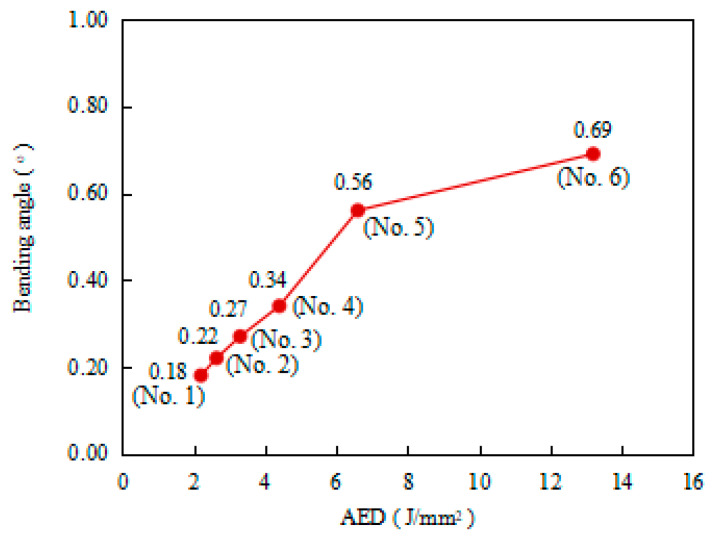
Bending angles of the plate at different AED values.

**Figure 5:**
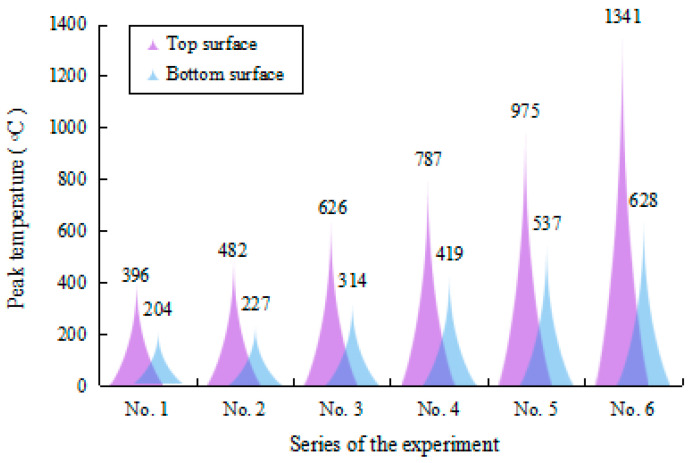
Peak temperature of the plate at different AED values.

**Figure 6:**
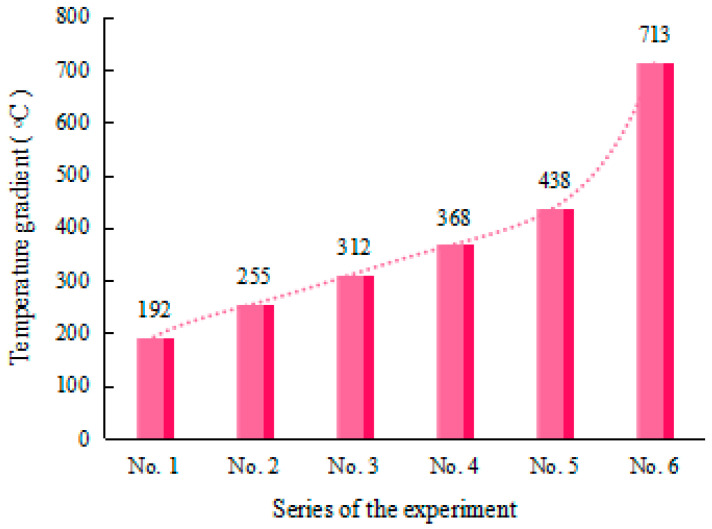
Temperature gradient of the plate at different AED values.

Due to the un-uniform nature of the bending angle along the laser scanning line [5], the average bending angle at different positions was employed to evaluate the bending angle of the plate. The average bending angle was calculated using the average of the bending angles measured at intervals of 20 mm along the scanning line, as shown in Figure 3. Figure 4 shows the average bending angles of the plate at different AED values, which are listed in Table 3. It is obvious that the bending angle increases with increasing AED value, which is in good agreement with the conclusions of Liu et al. [19]. This result is mainly due to the larger temperature gradient in the thickness direction at larger AED values. Figure 5 shows the peak temperature of the top and bottom surface of the plate, while Figure 6 presents the value of the temperature gradient of each case of the different AED values. From Figure 5, it can be seen that the peak temperatures of both the top and bottom surfaces of the plate increase with the increase in AED value. When the AED value increases from 2.2 J/mm^2^ (No. 1) to 13.2 J/mm^2^ (No. 6), the peak temperature of the top and bottom surface increases from 396 °C and 204 °C to 1341 °C and 628 °C, respectively. It can be found that the temperature gradient increases with increasing AED value. The larger temperature gradient induces more residual stress, which leads to a larger bending deformation.

However, it is also found from Figure 4 that the increase in the bending angle with increasing AED value is nonlinear. When the AED value is less than 6.6 J/mm^2^ (No. 1~5), the bending angle increase sharply, before it then slows down. This result may be attributed to the microstructure evolution of the material.

**Figure 7:**
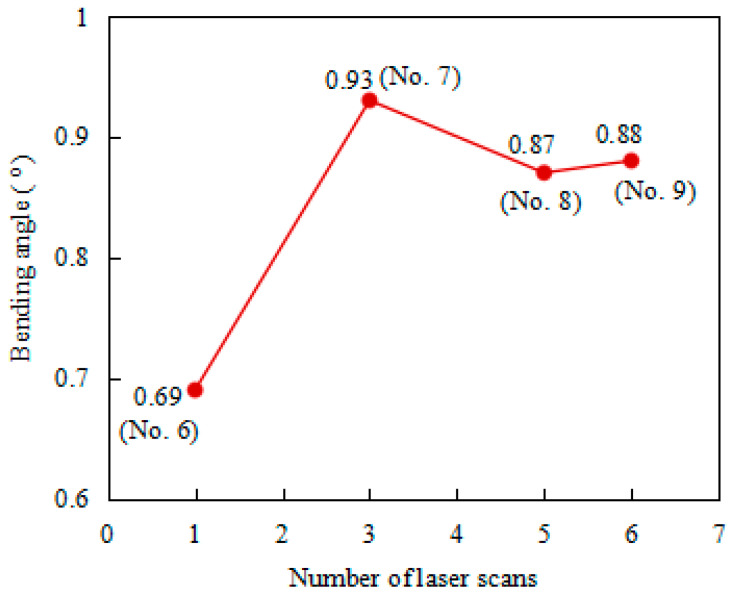
Bending angles of the plate at the same AED value.

**Figure 8:**
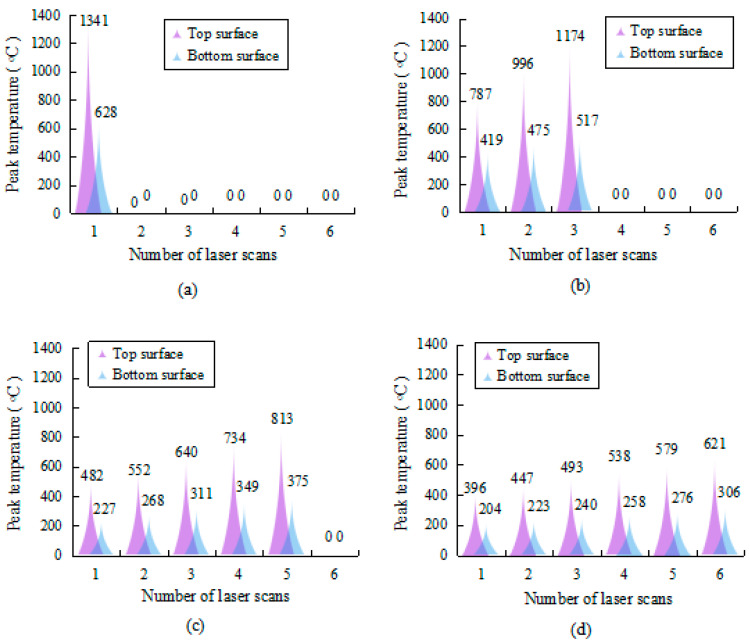
Peak temperature of the plate at the same AED value: (**a**) No. 6, (**b**) No. 7, (**c**) No. 8, and (**d**) No. 9.

**Figure 9:**
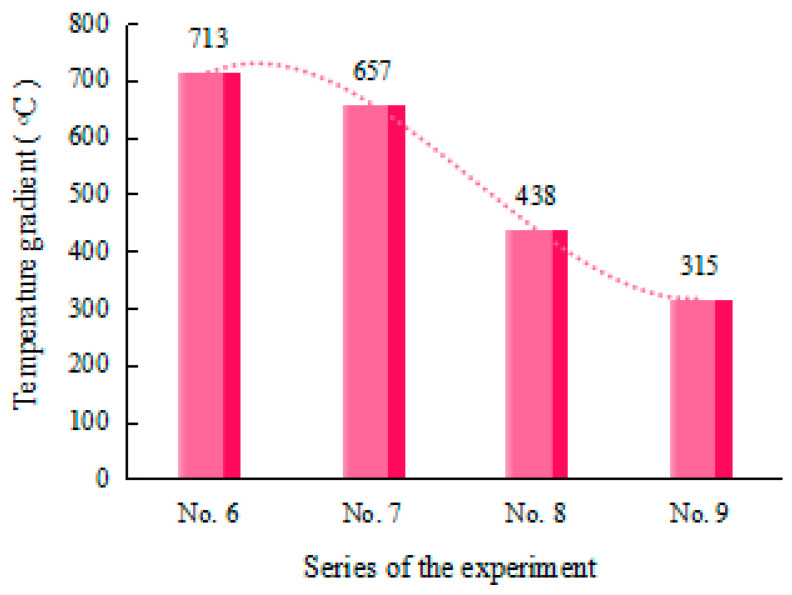
Temperature gradient of the plate at the same AED value.

The average bending angles of the plate at the same AED values as listed in Table 4 are shown in Figure 7. It can be seen that, under the four different laser scanning strategies, the bending angle of the single laser scan (No. 6) is the smallest (0.69°), while that of No. 7 has the largest bending angle (0.93°), which is an increase of 34.8% compared to No. 6. The bending angles of No. 8 and No. 9 are similar (0.87° and 0.88°, respectively), showing an increase of about 26% compared to No. 6. In order to analyze the reasons for this, the peak temperatures of the top and bottom surfaces of the plate and their temperature gradients in the four cases are investigated, as shown in Figure 8 and Figure 9.

From Figure 8, it is evident that, under the same AED conditions, the peak temperatures of the top and bottom surfaces decrease with an increase in the number of laser scans. When only one laser scan is conducted (No. 6), due to the slow scanning speed, the plate has a longer duration in which to absorb the laser energy, resulting in a significant increase in the temperature. The peak temperature of the top surface reaches up to 1341 °C, while that of the bottom surface reaches up to as high as 628 °C. When the laser scanning speed is gradually increased and the number of scans increased to six (No. 9), there is a considerable decrease in the peak temperatures of both surfaces of the plate, falling to 621 °C and 306 °C, respectively. This is due to the increased scanning speed, which results in less laser energy being absorbed by the plate per unit time and a better heat exchange with the surrounding air.

It is clearly visible in Figure 9 that, under the strategies of No. 6~9, the temperature gradient in the thickness direction decreases successively. That is, at the same AED value, the temperature gradient in the thickness direction decreases with an increase in the number of laser scans. However, the bending angle variation of the plate does not correspond with the change in the temperature gradient. This indicates that the temperature gradient of the plate is not the only factor that affects the bending angle. The microstructure evolution of the material may also play a significant role in affecting the bending behavior.

### 3.2. Microstructure Evolution

**Figure 10:**
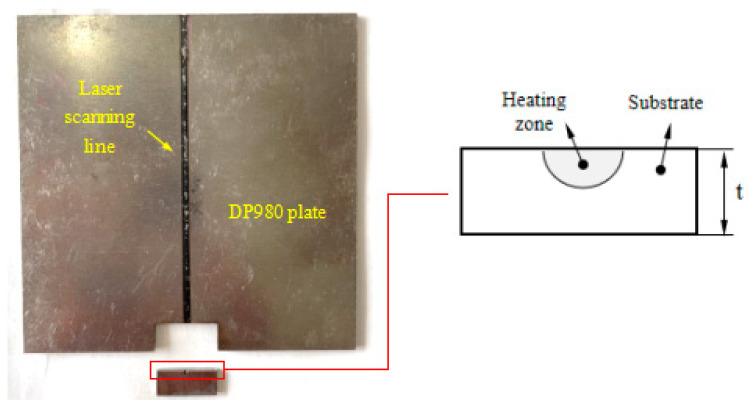
Schematic diagram of the microstructure observation areas.

Laser bending is a complex temperature–mechanical coupled deforming process, during which the microstructure of the DP980 material may change considerably. Figure 10 depicts the microstructure observation area on the cross-section of the laser scanning line. A scanning electron microscope (SEM) was employed to observe the microstructure evolution of the material.

**Figure 11:**
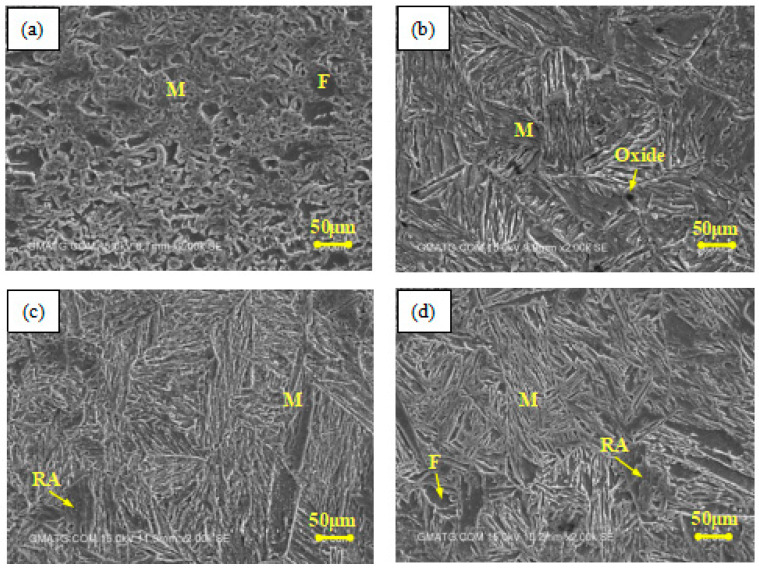
Microstructures of DP980 steel at different AED values: (**a**) substrate, (**b**) heating zone of No. 6, (**c**) No. 5, and (**d**) No. 4.

Figure 11 shows SEM images of material microstructures at the different AED values that are listed in Table 3. The microstructure of the DP980 substrate mainly consists of ferrite and martensite, as shown in Figure 11a. When the material is heated, as in the case of No. 6, the heating zone is full of martensite with a small amount of oxide, as shown in Figure 11b. The grains are coarse, which is due to the peak temperature (1341 °C) in this region being much higher than the Ac3 (917 °C) of the material. The grains grow rapidly during the laser heating process and most of the ferrite transforms into austenite, which further transforms into martensite during the cooling process. The increasing coarse grains largely reduce the plasticity and toughness of the material, which make plastic deformation difficult. Furthermore, due to the high strength of martensite, the increase in the martensite content makes the plastic deformation of the material more difficult. This is the main reason for the decrease in the bending angle growth rate of plate No. 6. From Figure 11c, it can be found that a small amount of residual austenite exists and the grains are fine. This is attributed to the peak temperature (975 °C) at which well-fined grains are obtained. However, due to the rapid cooling process, a small part of austenite cannot transform to martensite, but remains in the material. When the peak temperature of the heating zone is between Ac1 (721 °C) and Ac3 (No. 4), only parts of the ferrite transforms into austenite and then martensite. The microstructure of the material is not uniform, containing martensite, ferrite, and residual austenite, as shown in Figure 11d. When the peak temperature of the heating zone is under Ac1 (Nos. 1, 2, and 3), the microstructure of the material is almost unchanged. At this time, the bending angle of the plate mainly depends on the AED value of the material, which explains the reason for the nearly linear increase in the bending angle of specimen Nos. 1 to 3.

**Figure 12:**
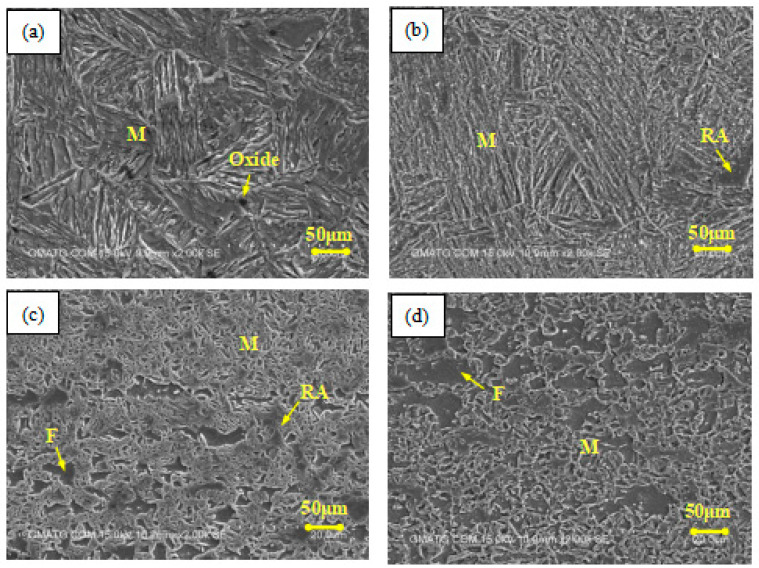
Microstructures of DP980 steel with the same AED value: (**a**) heating zone of No. 6, (**b**) No. 7, (**c**) No. 8, and (**d**) No. 9.

Figure 12 depicts the microstructures of the material at the same AED values that are listed in Table 4. It can be found that No. 7 is full of martensite, with a small amount of residual austenite; No. 8 consists of martensite, ferrite, and some residual austenite; and No. 9 mainly consists of martensite and ferrite, which is similar to the substrate. This is due to the fact that, when the material is heated under the strategy of No. 7, the peak temperature of the top surface rises gradually from 787 °C to 1174 °C, as shown in Figure 8b. The ferrite of the material transforms into austenite and then, during the cooling process, it mostly converts into uniform martensite, as shown in Figure 12b. Due to the homogenization of the martensite phase and the refinement of the grains, the plasticity of the material is improved, which leads to a larger bending angle of specimen No. 7 compared to No. 6. On the other hand, when the plate is processed under the strategy of No. 8, the peak temperature of the top surface rises with the number of laser scans in the range of 482~813 °C, as shown in Figure 8c. The ferrite grains undergo slight growth, and some of the ferrite begins to transform into austenite, resulting in an uneven microstructure, as shown in Figure 12c. This transformation reduces the plasticity of the material to some extent, which is the reason for the decrease in the bending angle of specimen No. 8 compared to No. 7. Fortunately, since the peak temperature is not too high (below Ac3) and does not cause a sharp growth of grains, the bending angle of specimen No. 8 is still significantly larger than that of No. 6. When the plate is scanned using the strategy of No. 9, the peak temperature of the top surface drops to the range of 396~621 °C, which is below Ac1. At this time, only a slight growth of the ferrite grains is observed, with no phase transformation occurring, as shown in Figure 12d. The bending behavior of the plate mainly depends on the AED value of the material, which is similar to that of specimen Nos. 1 to 3.

## 4. Conclusions

In this study, the bending angle variation of a DP980 steel plate under different laser scanning strategies is investigated. The microstructures of the material are analyzed, to explore the reason for the bending behavior. The main conclusions are summarized as follows:

(1) During a single laser scanning process, the bending angle of the plate increases with the increase in accumulated energy density (AED). When the peak temperature at the top surface of the plate is below Ac3, the bending angle increases almost linearly. But when it exceeds Ac3, the increment rate decreases.
(2) Under the same AED conditions, the bending angle of the plate undergoing multiple laser scans increases by at least 26% compared to the single scan.
(3) When the peak temperature at the top surface of the plate is between Ac1 and Ac3, the microstructure of the heating zone after cooling mainly consists of uniform martensite with some grain refinement; this is beneficial for the bending deformation of the plate. When the peak temperature is below Ac1, the microstructure changes in the heating zone are not significant and the bending efficiency of the plate is lower. However, when the peak temperature exceeds Ac3, the grains in the heating zone grow rapidly, which makes the bending deformation difficult and thereby causes a slow increase in the bending angle.

## Figures and Tables

**Table 1:** Chemical composition content of DP980 steel (mass fraction/%) [25].

C	Mn	Si	P	S	Al	Cr	Fe
0.19	2.07	0.13	0.01	0.001	0.05	0.1	Bal.

**Table 2:** Parameters of the laser system.

Brand	Model	Power (W)	Wavelength (nm)	Spot Diameter (mm)
IPG	YLR-150/750-QCW-AC	30~280	1070	2.5

**Table 3:** Laser scanning strategies at different AED values.

No.	Power (W)	Scanning Velocity (mm/s)	Number of Scans	AED (J/mm^2^)
1	220	30	1	2.2
2	220	25	1	2.64
3	220	20	1	3.3
4	220	15	1	4.4
5	220	10	1	6.6
6	220	5	1	13.2

**Table 4:** Laser scanning strategies at the same AED.

No.	Power (W)	Scanning Velocity (mm/s)	Number of Scans	AED (J/mm^2^)
6	220	5	1	13.2
7	220	15	3	13.2
8	220	25	5	13.2
9	220	30	6	13.2

## Data Availability

The original contributions presented in the study are included in the article, further inquiries can be directed to the corresponding author.
